# Brain MRI data in Chinese dyslexic children performing an auditory rhyming judgment task

**DOI:** 10.1016/j.dib.2017.02.029

**Published:** 2017-02-16

**Authors:** Fan Cao

**Affiliations:** Department of Communicative Sciences and Disorders, Michigan State University, United States

## Abstract

This article includes the description of data information from an auditory rhyming judgment task in Chinese children with developmental dyslexia, age-matched control children and reading-matched control children. You will find fMRI data information including experimental design, MRI protocol, and brain activation results from each of the three groups of subjects. Other results from the same study were published in Neuroimage (Cao, in press [1]).

**Specifications Table**

**Table:** 

Subject area	*Psychology*
More specific subject area	*fMRI of reading disability*
Type of data	*Table*
How data was acquired	*fMRI*
Data format	*Analyzed*
Experimental factors	*Children with developmental dyslexia, age-matched controls, reading-matched controls*
Experimental features	*Auditory word pairs were sequentially presented, and subjects were told to press a button if they rhyme and another button if they do not rhyme*
Data source location	*East Lansing, Michigan, USA*
Data accessibility	*Data are within this article*

Values of the data

• Current data can be used to examine the phonological processing in Chinese-speaking children.
• Current data can be used to compare to phonological processing in other languages.
• Current data can be used to compare to phonological processing in Chinese-speaking adults.
• Current data can be used to examine phonological processing in children with reading disability

## 1 Data

The fMRI data are from an auditory word rhyming judgment task in native Chinese speaking children with or without developmental dyslexia. Children with developmental dyslexia and age-matched control children were 5th-graders in elementary school with an age range of 10–12 years old. The reading matched control children were 3rd-graders in elementary school with an age range of 8–10 years old. The fMRI data were the result of brain activation in each of the three groups of subjects. Other results from this study were published in Neuroimage [1].

## 2 Experimental design, materials and methods

### 2.1 Cognitive tasks

#### 2.1.1 Auditory rhyming judgment

Two words were presented sequentially in the auditory modality and participants were asked to determine whether the second syllable of the words rhymed. All words consisted of two characters. In order to eliminate the possibility of making decisions based solely on phonological or tone information, we controlled for the similarity of the orthography, phonology, and tone of the second character in the first and the second word. There were 24 trials in each of 4 conditions, similar orthography and rhyming (O+P+, e.g. 弥补/bu3/, 纯朴/pu3/), similar orthography and non-rhyming (O+P-, e.g. 翻译/yi4/, 选择/ze2/), different orthography and rhyming (O-P+, e.g. 环保/bao3/, 大炮/pao4/), different orthography and non-rhyming (O-P-, e.g. 损坏/huai4/, 学科/ke1/). In half trials of each condition, the second character of the first and the second word had the same tone (e.g. 弥补/bu3/, 纯朴/pu3/), and in the other half they had different tones (e.g. 逮捕/bu3/, 胸脯/pu2/).

#### 2.1.2 Stimulus characteristics

All words used in this experiment did not have homophones. The two character words were matched on several variables across tasks, conditions, and presentation orders using analysis of variance (ANOVA) models of 2 task (rhyming and spelling)×4 condition (O+P+, O+P-, O-P+ and O-P-)×2 presentation order (first word and second word). These variables were adult written frequency (Beijing Language and Culture University, [2]), number of strokes, word familiarity in third-graders, and word familiarity in fifth-graders. Word familiarity was assessed in an independent study on 50 third-graders and 50 fifth-graders through a 7-point scale.

The second characters of words were also matched on several variables across tasks, conditions, and presentation orders using ANOVA models of 4 condition (O+P+, O+P-, O-P+ and O-P-)×2 presentation order (first word and second word). The variables were adult written frequency (Beijing Language and Culture University, [2]), and number of strokes.

#### 2.1.3 Control trials

For the perceptual control trials, three-tone auditory stimuli, where all the component tones were ranging from 300 to 875 Hz (in 25 Hz increments), were presented sequentially and the participant was asked to determine whether the tone pairs were identical. Each tone in the three-tone stimuli was 200 ms in duration with a 50 ms linear fade in and out. The perceptual control had 24 trials with half of them matched and half non-matched. There were also 48 null trials in which a black cross turned red indicating the need to press a button with the right index finger.

### 2.2 Experimental procedure

We used an event-related design with four 6 min 44 s runs for each subject including two runs. In each run, there were 12 s for a ‘dummy’ period at the beginning, and 22 s at the end in order to get the whole hemodynamic response function (HRF) for the last trial. In each run, there were 48 experimental trials, 12 perceptual control trials, and 24 null trials. For the experimental trials, the two spoken words were presented in sequential order and a black fixation-cross appeared throughout the trial. The duration of each word was between 500 and 800 ms, with the second word beginning 1000 ms after the onset of the first. A 2600 ms response interval occurred 800 ms after the onset of the second word. The start of the response interval was signified by a red fixation-cross and indicated the need to make a response. If the second syllable of the two words rhymed, the participant was asked to press a button with the index finger; if they did not rhyme, the participant was asked to press a different button with the middle finger. We presented the perceptual control trials in the same procedure as we did for the experimental trials. For 48 null trials, there was a black fixation cross (+) presented for 1800 ms and then a red cross was presented for 2600 ms indicating the need to press a button with the right index finger. Stimuli were presented in the same order for all subjects, optimized for random order using OptSeq (http,//surfer.nmr.mgh.harvard.edu/optseq).

### 2.3 MRI data acquisition

All images were acquired using a 3 T Siemens scanner. Gradient-echo localizer images were acquired to determine the placement of the functional slices. For the functional imaging studies, a susceptibility weighted single-shot EPI (echo planar imaging) method with BOLD (blood oxygenation level-dependent) was used. Functional images were interleaved from bottom to top in a whole brain EPI acquisition. The following scan parameters were used, TR=2000 ms, TE=20 ms, flip angle=80°, matrix size=128×128, field of view=220 mm, slice thickness=3 mm, number of slices=33. These scanning parameters resulted in a 1.7×1.7×3 mm voxel size. At the end of the functional imaging session, a high resolution, T1 weighted 3D image was acquired (MPRAGE, TR=2390 ms, TE=2.9 ms, TI=900 ms, flip angle=20°, matrix size=256×256, field of view=256 mm, slice thickness=1 mm, number of slices=160). The orientation of the 3D volume was identical to the functional slices.

### 2.4 Image data analysis

Data analysis was performed using SPM8 (Statistical Parametric Mapping) (http,//www.fil.ion.ucl.ac.uk/spm). The functional images were corrected for differences in slice-acquisition time to the middle volume and are realigned to the last volume in the scanning session using affine transformations. No individual runs had more than 4 mm maximum movement for any subject in the *x*-plane, *y*-plane or *z*-plane. Furthermore, no individual runs had more than 3° of maximum displacement in rotation for pitch, yaw or roll. All statistical analyses were conducted on these movement-corrected images. Co-registered images were normalized to the MNI (Montreal Neurological Institute) average template (12 linear affine parameters for brain size and position, 8 non-linear iterations and 2×2×2 nonlinear basis functions). Statistical analyses were calculated on the smoothed data (4×4×8 mm isotropic Gaussian kernel).

**Fig. 1:**
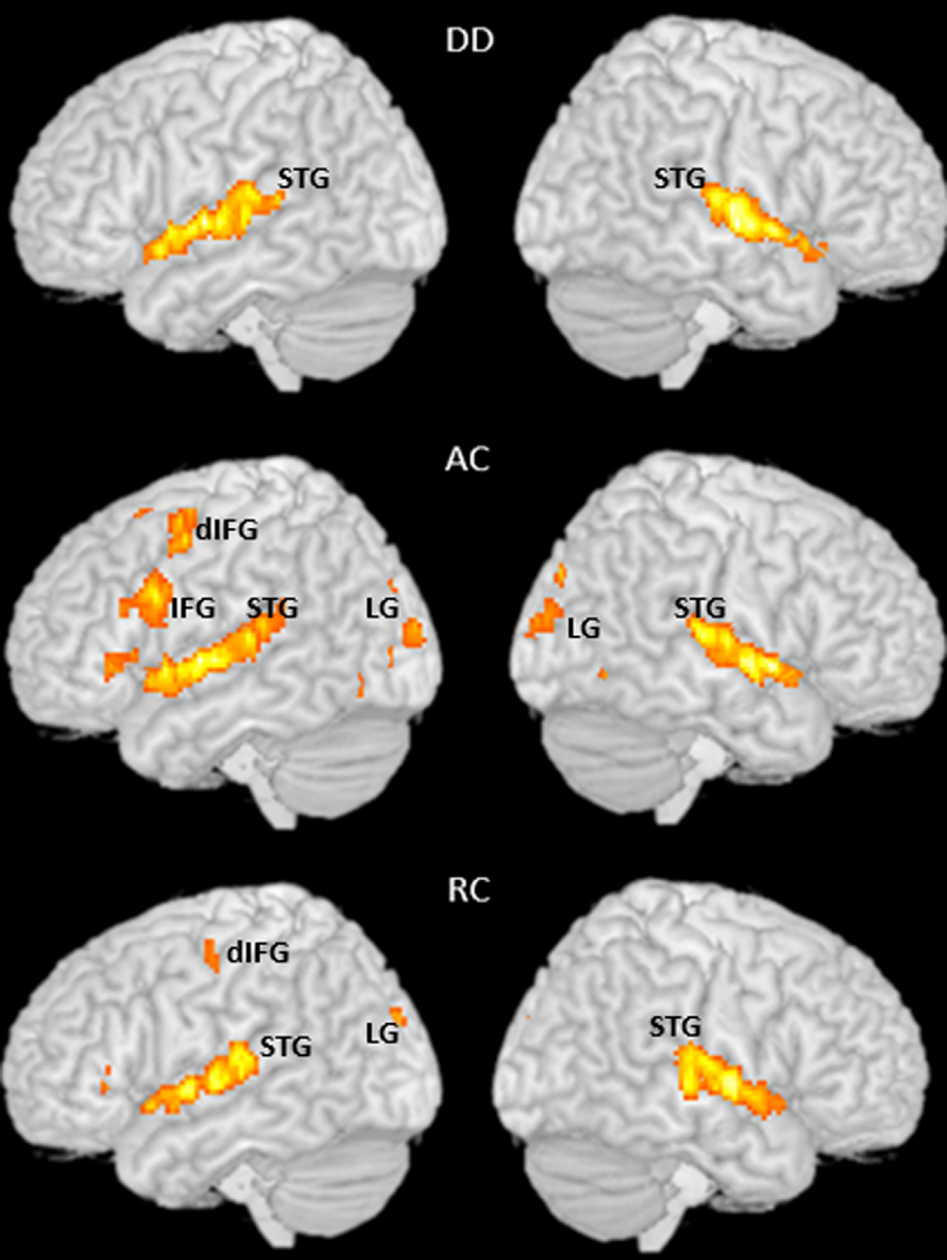
Brain activation during the auditory rhyming task for the DD, AC and RC group. STG: superior temporal gyrus; IFG: inferior frontal gyurs; dIFG: dorsal inferior frontal gyrus; LG: lingual gyrus.

Data from each subject were entered into a general linear model using an event-related analysis procedure. Word pairs were treated as individual events for analysis and modeled using a canonical HRF. Statistics was calculated with a high pass filter (128 s cutoff period). We used global normalization to scale the mean of each scan to a common value. Parameter estimates from contrasts of the canonical HRF in single subject models were entered into random-effects analyses. All reported results were at a threshold of FWE corrected *P*<.05, greater than 20 voxels (Table 1, Fig. 1).

## Figures and Tables

**Table 1:** Brain Activation in the auditory rhyming judgment task in children with developmental dyslexia, age-matched controls and reading-matched controls.

	Anatomical region	H	BA	Voxels	x	*y*	*z*	*Z*
Children with developmental dyslexia								
Superior temporal gyrus		R	21/22	910	56	−16	−2	7.47
Superior temporal gyrus		L	22	814	−62	−8	0	7.31
Right calcarine fissure		R	18, 19	37	26	−56	4	5.60
Culmen		L	–	59	−10	−54	−2	5.38
Cuneus		L	18	36	−2	−82	16	5.37

Age-matched controls								
Middle temporal gyrus, superior temporal gyrus		R	22	877	64	0	−4	7.57
Superior temporal gyrus		L	22	769	−62	−8	0	7.39
Lingual gyrus		R	18	1822	26	−62	0	6.86
Supplemental motor area		L/R	6/8	274	0	16	46	6.75
Inferior frontal gyrus		L	9/46	266	−46	10	26	6.63
Precentral gyrus		L	6	66	−50	−2	48	6.21
Superior temporal gyrus		L	22	181	−60	−34	14	6.15
Insula		L	–	84	−30	26	0	6.07
Middle frontal gyrus		L	6	43	−36	0	56	6.05
Cuneus		R	19	24	8	−86	34	5.89
Thalamus		L	–	40	−12	−16	6	5.89
Globus pallidus		L	–	33	−16	0	−2	5.74
Hippocampus		L	–	26	−18	−28	−6	5.73
Middle frontal gyrus		L	6/8	36	−24	2	52	5.64
Middle occipital gyrus		R	18	64	16	−92	14	5.54
Cuneus		L	19	21	−4	−84	30	5.54

Reading-matched controls								
Superior temporal gyrus, middle temporal gyrus		R	22, 21	1034	48	−10	0	7.19
Superior temporal gyrus		L	22	643	−54	−16	0	6.93
Superior temporal gyrus		L	22,42	133	−62	−26	6	6.59
Lingual gyrus		L	18	180	−12	−60	2	6.29
Insula		L	–	30	−28	28	2	5.90
Cuneus		L	19	23	−6	−90	26	5.76
Lingual gyrus		L	19	28	−20	−66	−4	5.64
Medial frontal gyrus		L	6	20	−2	10	52	5.61
Lingual gyrus		R	18	28	20	−56	0	5.48
Lingual gyrus		R	18	21	12	−70	−8	5.45
Cuneus		L	18	22	−2	−80	12	5.39
Precentral gyrus		L	6	25	−44	−14	56	5.35
